# Constitutive Active CPK30 Interferes With Root Growth and Endomembrane Trafficking in *Arabidopsis thaliana*

**DOI:** 10.3389/fpls.2022.862398

**Published:** 2022-06-16

**Authors:** Ren Wang, Ellie Himschoot, Jian Chen, Marie Boudsocq, Danny Geelen, Jiří Friml, Tom Beeckman, Steffen Vanneste

**Affiliations:** ^1^Department of Plant Biotechnology and Bioinformatics, Ghent University, Ghent, Belgium; ^2^VIB Center for Plant Systems Biology, Ghent, Belgium; ^3^Université Paris-Saclay, CNRS, INRAE, Univ. Evry, Institute of Plant Sciences Paris-Saclay (IPS2), Orsay, France; ^4^Université de Paris, Institute of Plant Sciences Paris-Saclay (IPS2), Orsay, France; ^5^Department of Plants and Crops, Ghent University, Ghent, Belgium; ^6^Institute of Science and Technology Austria, Klosterneuburg, Austria; ^7^Lab of Plant Growth Analysis, Ghent University Global Campus, Incheon, South Korea

**Keywords:** calcium-dependent kinase, CPK30, endosome, Brefeldin A, PIN, root, gravitropism, polarity

## Abstract

Calcium-dependent protein kinases (CPK) are key components of a wide array of signaling pathways, translating stress and nutrient signaling into the modulation of cellular processes such as ion transport and transcription. However, not much is known about CPKs in endomembrane trafficking. Here, we screened for CPKs that impact on root growth and gravitropism, by overexpressing constitutively active forms of CPKs under the control of an inducible promoter in *Arabidopsis thaliana*. We found that inducible overexpression of an constitutive active CPK30 (CA-CPK30) resulted in a loss of root gravitropism and ectopic auxin accumulation in the root tip. Immunolocalization revealed that CA-CPK30 roots have reduced PIN protein levels, PIN1 polarity defects and impaired Brefeldin A (BFA)-sensitive trafficking. Moreover, FM4-64 uptake was reduced, indicative of a defect in endocytosis. The effects on BFA-sensitive trafficking were not specific to PINs, as BFA could not induce aggregation of ARF1- and CHC-labeled endosomes in CA-CPK30. Interestingly, the interference with BFA-body formation, could be reverted by increasing the extracellular pH, indicating a pH-dependence of this CA-CPK30 effect. Altogether, our data reveal an important role for CPK30 in root growth regulation and endomembrane trafficking in *Arabidopsis thaliana*.

## Introduction

Calcium (Ca^2+^) is one of the most conserved second messengers in living organisms, and is a central component of signal transduction networks mediating plant development and responses to numerous biotic and abiotic stresses (Shi et al., 2018), such as pathogens, drought, heat, salinity and cold, etc. A wide variety of endogenous and external stimuli can activate cytoplasmic Ca^2+^ signals that can be sensed and decoded by multiple classes of Ca^2+^ binding proteins that provide specificity in the signaling pathway: the calmodulins (CaM), the calcineurin B-like (CBL) proteins that mostly regulate the class of CBL-interacting protein kinases (CIPKs) in plants and the calcium-dependent protein kinases (CPKs) and their relatives CPK-related kinases (CRKs) (Yip Delormel and Boudsocq, 2019). CaM is highly conserved in all eukaryotes, whereas CBLs and CPKs are only identified in plants and some protists (Day et al., 2002; Shi et al., 2018).

CPKs are serine/threonine protein kinases with an auto-inhibitory junction, and calmodulin-like domain in a single polypeptide. As a result, CPKs can be activated by direct binding of Ca^2+^ in its calmodulin-like domain (Cheng et al., 2002; Roberts and Harmon, 2003), and constitutive active variants can be generated by deleting the auto-inhibitory and calmodulin-like domains (Boudsocq et al., 2010; Supplementary Figure 1A). The Arabidopsis genome contains 34 CPK genes, operating in a diverse range of signaling pathways related to plant immunity, abiotic stress, hormonal signaling, regulation of the cytoskeleton, ion and water transport, nitrogen, and phospholipid metabolism (Rudd and Franklin-Tong, 2001; Boudsocq and Sheen, 2013; Simeunovic et al., 2016; Yip Delormel and Boudsocq, 2019). While some have a ubiquitous expression in most tissues, others are only expressed in specific tissues (Hong et al., 1996; Ye et al., 2009; Monaghan et al., 2014).

Several CPKs are also known to be involved in root development. A subclade of CPKs, including CPK10, 30, 32, have been implicated in a nitrate signaling cascade that controls root architecture (Liu et al., 2017). These CPKs modulate the function of important regulatory factors of the nitrate signaling pathway such as the transcription factor NIN-LIKE PROTEIN 7 (NLP7), presumably downstream of the transceptor NPF6.3/NRT1.1 (Liu et al., 2017). The CPK7 acts on root hydraulic conductivity by reducing the cellular abundance of water transporting aquaporins (Li et al., 2015). CPK3 was linked to lateral root formation by *in vitro* phosphorylation of the C-terminus of PLAIV (Rietz et al., 2010). Several calcium-dependent protein kinases (CPKs), including CPK4, can phosphorylate RopGEF1 to promote its degradation in root hair development (Li et al., 2018). Interestingly, CPK29 was found to impact on auxin-regulated development through modulation of auxin-transport by directly phosphorylating of PIN-type auxin transporters (Lee et al., 2021).

A plant cell, as well as any other eukaryotic cell, contains a network of intracellular membranes to facilitate protein transport, which consists of the plasma membrane, the ER, *trans*-Golgi network/early endosomes (TGN/EEs), the Golgi Apparatus, the multivesicular body/prevacuolar compartment/late endosomes (MVB/PVC/LEs), and the lytic and storage vacuole (Murphy et al., 2005; Jürgens and Geldner, 2007; Schellmann and Pimpl, 2009; Zárský and Potocký, 2010; Reyes et al., 2011; Robinson and Pimpl, 2014). Key regulators of endosome trafficking between distinct endomembrane compartments are ARF (ADP-ribosylation Factor) GTPases and their regulators ARF-GEFs (ARF-GUANINE NUCLEOTIDE EXCHANGE FACTORs) (Zárský and Potocký, 2010; Kania et al., 2014).

ARF GTPases control the budding of endosomal vesicles, and continuously cycle between a guanosine-5′-triphosphate (GTP)-bound (active) form, and a guanosine diphosphate (GDP)-bound (inactive) form. Switching between both forms relies on GTP hydrolysis, mediated by negative regulators named ARF GTPase-ACTIVATING PROTEINs (ARF-GAPs), and exchange of GDP for GTP, mediated by the positive regulators called ARF-GEFs (Yorimitsu et al., 2014). The Arabidopsis genome contains 8 large ARF-GEFs showing mutual significant functional redundancy (Anders and Jurgens, 2008). Due to the presence of several resistant ARF-GEFs in Arabidopsis, the fungal toxin Brefeldin A can be used to inhibit GNOM ARF-GEF-regulated processes in the root. At a concentration of 25 μM, BFA induces the formation of so-called BFA bodies that are aggregates of early endosomes/TGN surrounded by Golgi (Donaldson and Jackson, 2000; Geldner et al., 2001; Nebenfuhr et al., 2002), an effect that can be largely attributed to inhibition of GNOM (Peyroche et al., 1996; Geldner et al., 2003). In these BFA bodies, *de novo* synthesized and endocytosed cargoes destined for secretion and recycling, respectively, can be captured. Therefore, the accumulation of endosomal cargoes could be used as a proxy for endocytic rates (Geldner et al., 2001; Robert et al., 2010; Kitakura et al., 2011). However, caution should be taken when using this assay, as treatments that interfere with BFA-induced endosomal aggregation could complexify the interpretation of results (Narasimhan et al., 2021).

While CPKs have been found in various subcellular locations, most CPKs are targeted to the plasma membrane (PM), where they modulate the activity of PM-localized proteins such as ion channels like SLAH (Gutermuth et al., 2013) and transporters (Liu et al., 2017; Lee et al., 2021). However, not much is known about their effect on endomembrane trafficking (Himschoot et al., 2017). Here, we screened constitutive active CPKs for effects on root growth, and found that CPK30 and the related CPK13 strongly impair root growth and gravitropism. This phenotype was associated with defects in endosomal trafficking, and was pH dependent. These data provide a first link between CPK activity and the regulation of endomembrane dynamics.

## Materials and Methods

### Plant Growth Conditions

*Arabidopsis thaliana* seeds were sterilized by using bleach gas (8 mL concentrated HCl to 150 mL bleach) overnight, afterward the seeds were sown on Petri dishes (12 cm × 12 cm) containing sterile half-strength Murashige and Skoog (1/2 × MS, Duchefa-biochemie, Haarlem) medium (1/2 × MS salts, 0.8% sucrose, 0.5 g/L 2-(N-morpholino) ethanesulfonic acid, pH 5.7, and 1% w/v agar), after 2 days stratification at 4°C in the dark then transfer to growth chamber. Plates are put vertically at 21°C under continuous light. The phenotype of Col-0, *CA-CPK* lines was determined by germinating seeds on 1/2 x MS, and transferring them after 5 days 1/2 × MS plates supplemented with 2.5 μM β-estradiol for another 7 days. CA-CPK30 was crossed to DR5rev:GFP (Friml et al., 2003) and analyzed in the F1 generation.

### Cloning and Selection

The CA-CPK clones were previously created for transient expression assays (Boudsocq et al., 2010). These vectors were used as templates for subcloning in pDONR221 *via* BP reaction of a PCR fragments that were generated using attB1_CPKx_FW (GGGGACAAGTTTGTACAAAAAAGCAGGCTTGATCGTGC AACCCATCGGATCC) and AttB2_FLAG_REV (GGGGACC ACTTTGTACAAGAAAGCTGGGTTCACTTGTCATCGTCGT CC). The resulting entry vectors were confirmed by sequencing and recombined with pEN-L4_RPS5A_XVE_R1 (Gao et al., 2018) into pH7m24GW,3 (Karimi et al., 2007) *via* LR recombination. Validated clones were transformed into wild-type Col-0 plants *via* floral dip transformation (Clough and Bent, 1998), and transformants were selected using Hygromycin selection.

### Chemicals

The following chemicals were used: Brefeldin A (BFA, catalog Nr B6542-25MG-Sigma-Aldrich, Belgium), β-estradiol (catalog Nr E8875-5G-Sigma-Aldrich, Belgium). These drugs were dissolved in 100% dimethylsulfoxide (DMSO, catalog Nr D4540-500ML Sigma-Aldrich, Belgium) to make 50 mM stock solutions. The FM4-64 (T13320—Thermo Fisher Scientific, Belgium) stock in DMSO was set at 10 mM.

### Immunodetection

The seedlings used for immunodetection are 3–4-day-old grown on 1/2 × MS, and then treated in liquid medium as indicated. For β-estradiol induction, 3-day-old seedlings were transferred to a plate 1/2 × MS medium containing β-estradiol (2.5 μM) for at least 24 h. The samples were fixed by paraformaldehyde (4%) (cat. no. P6148, Sigma-Aldrich, Belgium) in PBS for 1 h in vacuum. The following steps of the immunostaining were performed by the immuno-robot InsituPro Vsi II (Intavis, Tübingen, Germany), as described by Sauer (Sauer et al., 2006). In brief, immediately after fixation, the samples were transferred to the 30-well rack of the robot and subjected to 6 washes [3 in PBS pH 7.4 + 0.1%Triton X-100 (PBS-T); 3 in distilled water + 0.1%Triton X-100 (cat. no. 3051.2, Carl Roth, Germany); 5 min each], cell wall digestion [1.5% Driselase (cat. no. D9515, Sigma-Aldrich, Belgium) in PBS, 30 min at 37°C], 3 washes (PBS-T, 5 min), permeabilization (3% IGEPAL CA-630 (cat. no. I3021, Sigma-Aldrich, Belgium), 10% DMSO in PBS; 30 min room temperature), 3 washes (PBS-T, 5 min), blocking solution (3% BSA in PBS, 1 h 37°C), primary antibody solution (primary antibodies diluted in blocking solution, 4 h 37°C), 5 washes (PBS-T, 5 min), secondary antibody solution (secondary antibodies diluted in blocking solution, 3 h, 37°C), 5 washes (PBS-T, 5 min) and 5 washes (distilled water, 5 min). The dilutions of the primary antibodies used are: goat anti-PIN1 (1:600) (sc-27163, SantaCruz, CA, United States), rabbit anti-PIN1 (1:1,000) (Paciorek et al., 2005) and rabbit anti-PIN2 (1:1,000) (Abas et al., 2006). The dilutions of the secondary antibodies used are: AlexaFluor488 donkey anti-goat (1:600) (A-11055, Thermo Fisher Scientific, Belgium) and Alexafluor555 donkey anti-rabbit (1:600) (A-31572, Thermo Fisher Scientific, Belgium).

### Microscopy and Image Analysis

A Leica SP2 or a Zeiss LSM 710 confocal laser scanning microscope equipped with a 63x water-corrected objective (Leica SP2) or 40x water-corrected objective (Zeiss LSM710) was used for detection. Settings for detection: Alexa488 (ex 488 nm/em 500–545 nm), Alexa555 (ex 561 nm/em 555–610 nm), the pinhole was always set to 1 airy unit. Images were analyzed using Fiji^1^: Eg. Root length (segmented line for tracing the root, Analyze-Measure (Analyze-set scale for calibration); Fiji was used to rotate (Image-Transform-Rotate) and crop images (Image-Crop). For the maximal projections: Stacks-z-Project + Image-Lookup Tables-Fire. Fluorescence measurements were done on 8-bit images. The proportion of cells with BFA bodies was scored manually and calculated by using Excel. BoxPlotR was used to generate the box plots (Spitzer et al., 2014).

### Statistical Analysis

For statistical analysis of the immunolocalization experiments, a logistic regression was performed to compare the presence of BFA bodies in root cells of treated vs. untreated roots or wild type vs. mutant. A random effect was added to the model for the experiments with multiple repeats to consider the correlation between measurements done at the same time. The analysis was performed with the glimmix procedure from SAS (Version 9.4 of the SAS System for windows 7 64 bit. Copyright 2002–2012 SAS Institute Inc. Cary, NC, United States).^2^ Maximum likelihood estimation was done with the default estimation method. A Wald-type test was performed to estimate the treatment/genotype effect on the presence of BFA bodies in the root cells.

## Results

### A Gain-of-Function Screen Identifies Calcium-Dependent Protein Kinases Involved in Auxin-Regulated Root Growth

**FIGURE 1:**
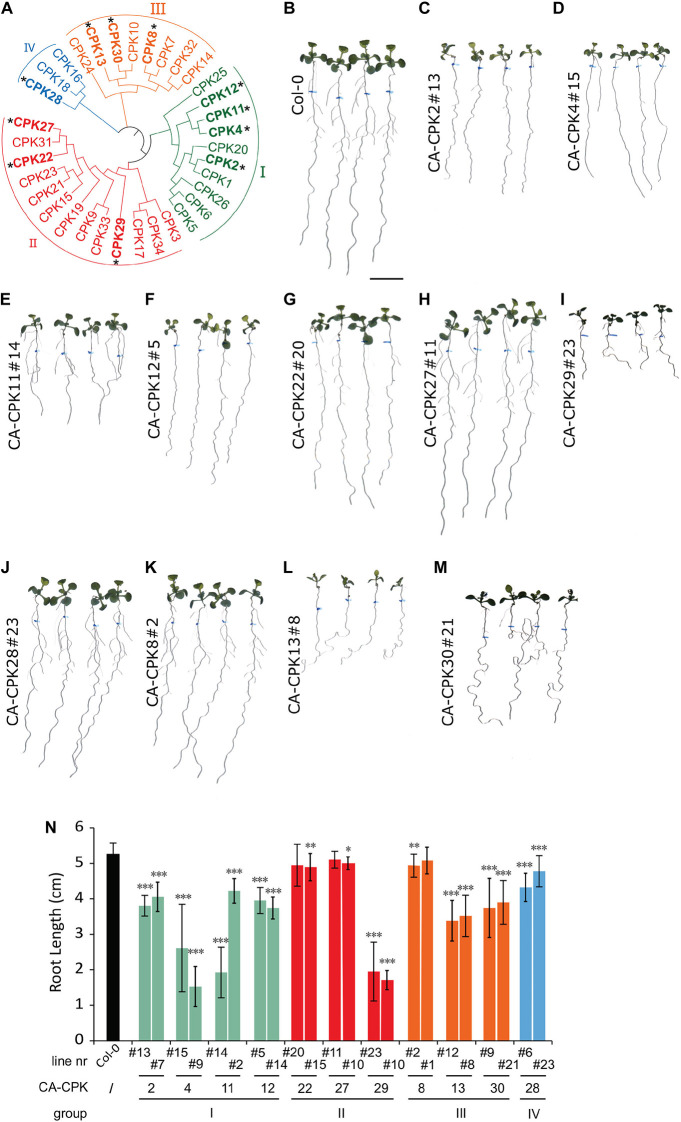
Phenotypic screen of constitutive active CPKs. **(A)** Phylogenetic tree of the Arabidopsis CPK family, with indication of the four main subgroups (I-IV). CPKs indicated with an asterisk were analyzed *via* stable overexpression lines. **(B–M)** Overview of macroscopic phenotypes of CA-CPK lines relative to WT (Col-0). Seeds were grown for 5 days on 1/2 × MS medium then transferred to medium supplemented with 2.5 μM β-estradiol for another 7 days. Scale bar = 1 cm. **(N)** Quantification of root length for indicated CA-CPK lines. Data are represented as the mean ± SD of at two independent replications. *n* ≥ 12 for all lines, except for CPK12#14 (*n* = 6) and CA-CPK22#20 (*n* = 7). Asterisk indicates significant difference (Unpaired Student’s *t*-test; *P ≤ 0.05, ^**^P ≤ 0.01, ^***^P ≤ 0.001) between transgenic lines and WT (Col-0) plants.

A gain-of-function screen was performed to identify CPKs involved in root growth and gravitropism. Therefore, we generated a collection of β-estradiol-inducible vectors for inducible overexpression of constitutive active (CA) variants, lacking the C-terminal Ca^2+^ regulatory and autoinhibitory domain, for 12 out of 34 CPKs in *Arabidopsis* (Figure 1A and Supplementary Figure 1A). The templates for cloning the CA-CPKs were previously used for transient expression in a protoplast system (Boudsocq et al., 2010; Liu et al., 2017), and the β-estradiol induction module was driven under the RPS5A promotor (Gao et al., 2018). We obtained at least 2 independent lines for Group I: CA-CPK2, CA-CPK4, CA-CPK11, CA-CPK12; Group II: CA-CPK22, CA-CPK27, CA-CPK29; Group III: CA-CPK8, CA-CPK13 and CA-CPK30; Group IV: CA-CPK28, and screened them at homozygosity for obvious β-estradiol-induced root phenotypes (Figures 1B–M and Supplementary Figures 1B–M). Seeds were germinated on control medium and transferred after 5 days to β-estradiol medium (2.5 μM) for another 7 days. The position of the root tip at time of transfer was marked to allow measuring the root growth during estradiol treatment. The β-estradiol treatment significantly reduced root growth in both independent lines for CA-CPK2, CA-CPK4, CA-CPK11, CA-CPK12, CA-CPK13, CA-CPK29, and CA-CPK30 (Figure 1N). The observation of root growth defects in 2 independent lines illustrates robustness of the observed phenotypes, and highlights the corresponding CPKs as regulators of processes that are important for root growth.

### Constitutive Active CPK30 Interferes With Auxin Distribution and Gravitropism

**FIGURE 2:**
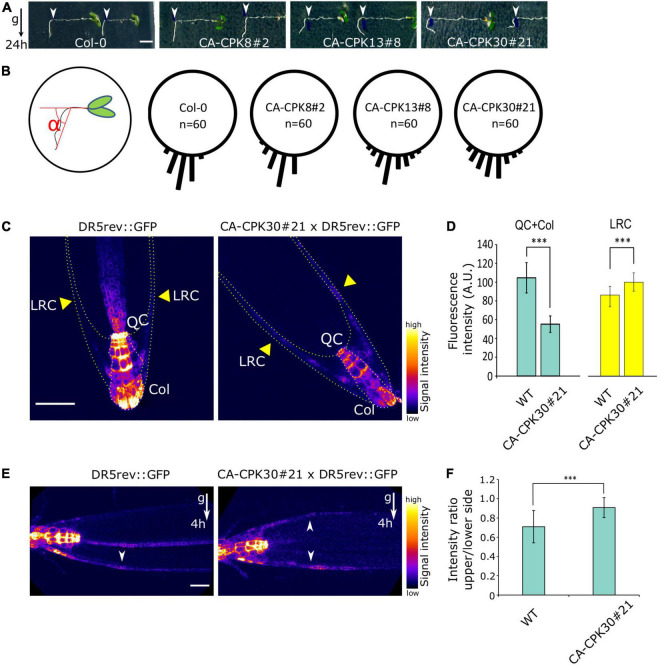
Induction of CA-CPK30 causes agravitropic root growth. **(A)** Analysis of the root gravitropic response of Col-0, CA-CPK8#2, CA-CPK13#8 and CA-CPK30#21. 5-day-old seedlings were transferred to 2.5 μM β-estradiol medium and immediately gravistimulated for 24 h (90 degree rotation). White arrowheads mark the position of the root tip at the moment of transfer. Scale bar = 0.2 cm. **(B)** Quantification of the root angle distribution of **(A)**. *n* = 60 is the total number of roots analyzed from two replicates for each genotype. **(C)** Expression pattern of the auxin output response marker DR5rev::GFP in WT and CA-CPK30#21 backgrounds. 6-day-old seedlings were transferred to 2.5 μM β-estradiol medium for a 2 day induction. Yellow arrowheads indicate the lateral root cap (LRC). QC and Col, are quiescence center and columella, respectively. Scale bar = 50 μm. **(D)** Quantification of DR5rev::GFP fluorescence in WT and CA-CPK30#21 (shown in **C**), in the quiescence center and columella, lateral root cap. Measured areas are indicated in **(C)** by dashed lines. For DR5rev::GFP *n* = 12 in total, for CA-CPK30#21 × DR5rev::GFP *n* = 10 in total, from two replicates. Data are presented as the mean ± SD. (Unpaired Student’s *t*-test; ^***^P ≤ 0.001). **(E)** Expression pattern of the auxin output response marker DR5rev::GFP in WT and CA-CPK30 in response to gravity. 6-day-old seedlings were transplanted onto 2.5 μM β-estradiol-containing medium for 2 day induction, followed by a 4 h gravistimulation (90 degree rotation). Scale bar = 20μm. **(F)** Quantification of intensity ratio between the upper/lower side for DR5rev::GFP fluorescence in WT and CA-CPK30#21 (shown in **E**). Across 3 replicates, *n* = 17 for DR5rev::GFP and, *n* = 24 for CA-CPK3#21 × DR5rev::GFP. Data are presented as the mean ± SD. (Unpaired Student’s *t*-test; ^***^P ≤ 0.001).

In addition to a root length reduction, we noted that both lines of the closely related CA-CPK13 and CA-CPK30 displayed an exaggerated wavy root growth phenotype that was not seen for other CA-CPKs (Figures 1L,M and Supplementary Figures 1L,M). We further explored the root gravitropic growth in CA-CPK13#8 and CA-CPK30#21. Five-day-old seedlings were transferred to β-estradiol-containing medium and were rotated 90 degrees relative to the gravity vector followed by the assessment of gravitropic reorientation of the root tip after 24 h. As controls we used WT (Col-0) and CA-CPK8#2 that represents a more distant member of the group III CPKs, to which also CPK13 and CPK30 belong. In contrast to the tight realignment to the gravity vector of these controls, the realignment of CA-CPK13#8 and CA-CPK30#21 roots appeared to occur randomly, indicating a defect in their gravitropic response (Figures 2A,B). These data suggest that CA-CPK13 and CA-CPK30 deregulate processes that are important for normal root growth and gravitropism.

Gravitropism is the result of stimulation and inhibition of cell elongation driven by differential accumulation of auxin on opposing sides of a gravistimulated root (Vanneste and Friml, 2009). Therefore, we suspected a defective auxin distribution in the roots of CA-CPK30. We crossed the auxin signaling output marker DR5rev::GFP to CA-CPK30#21, yielding a gravitropic root defect similar to the one seen in the original CA-CPK30#21 (Supplementary Figures 2A,B), suggesting that the DR5rev::GFP background did not modify CA-CPK30 effects on gravitropism.

Subsequently, we analyzed the effect of 2 days β-estradiol treatment on the DR5rev::GFP expression pattern in the root tip. In the WT, DR5rev::GFP was mainly expressed in the quiescence center (QC) and columella (Col), with little to no expression in the lateral root cap (LRC) (Figure 2C). In the CA-CPK30, the QC and Col signals were reduced, while its intensity in the LRC increased (Figures 2C,D).

Next, we assessed the response of the DR5rev::GFP signal to a gravistimulus. Seedlings were transferred to β-estradiol for 2 days, followed by a 4 h gravistimulation. The gravistimulus elicited a typical asymmetric DR5rev::GFP expression at the lower side of the gravistimulated root meristem, but not at its upper side (Figures 2E,F). This reflects a differential auxin accumulation that inhibits elongation on the lower side, to achieve root bending and realignment to the gravitropic field. In contrast, the establishment of an asymmetric DR5rev::GFP signal between the upper and lower sides of CA-CPK30#21 was impaired (Figures 2E,F). These data suggest that CA-CPK30 causes gravitropic defects in the root *via* ectopic auxin accumulation that is unresponsive to gravistimulation.

### Constitutive Active CPK30 Impairs PIN Protein Levels and Polarity

**FIGURE 3:**
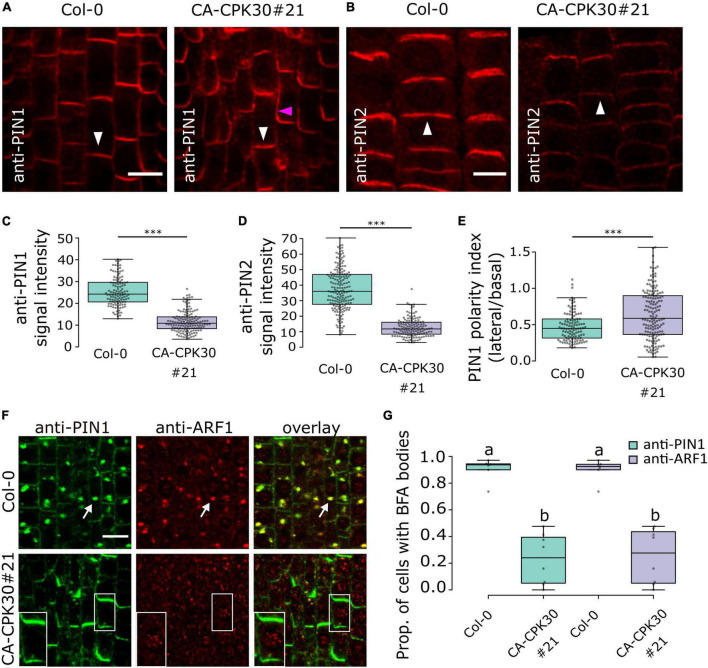
Induction of CA-CPK30 causes endomembrane trafficking defects. **(A,B)** Immunolocalization of PIN1 **(A)** and PIN2 **(B)** in roots of β-estradiol-treated Col-0 and CA-CPK30#21. White arrowheads indicate the expected polar domain of the respective PINs. The pink arrowhead indicates ectopic, lateral PIN1 signal. Scale bar = 20 μm. **(C)** Quantification of anti-PIN1 signal intensity in the plasma membrane of stele cells for Col-0 and CA-CPK30#21. For Col-0 *n* = 119 cells from 11 roots, for CA-CPK30#21 *n* = 147 cells from 9 roots pooled from two replicates. **(D)** Quantification of anti-PIN2 intensity in the plasma membrane of epidermal cells for Col-0 and CA-CPK30#21. For Col-0 *n* = 183 cells from 12 roots, for CA-CPK30#21 *n* = 140 cells from 11 roots pooled from two replicates. **(E)** Quantification of PIN1 polarity, based on the ratio of the PIN1 signal in the lateral/basal side for individual cells in Col-0 and CA-CPK30#21. For Col-0: *n* = 113 cells from 11 roots, for CA-CPK30#21: *n* = 158 cells from 10 roots, two replicates. (Unpaired Student’s *t*-test; *** *P* ≤ 0.001 for **C–E**). **(F)** Whole-mount immunolocalization using anti-PIN1 and anti-ARF1 antibodies in 5 day-old seedling root meristems of BFA-treated Col-0 and CA-CPK30#21 seedlings. Seedlings were transferred for 1 day to 2.5 μM β-estradiol prior to 1 h BFA (25 μM) treatment. White arrows indicate PIN1, ARF1 co-localization in BFA bodies. Insets are magnifications of a single cell of the BFA-treated CA-CPK30#21. Scale bar = 10 μm. **(G)** Boxplot representation of the proportion of cells with BFA bodies for treatments to Col-0 and CA-CPK30#21 (for Col-0: *n* = 6 and for CA-CPK30#21: *n* = 8 in total from two replicates). Significant differences (*P* ≤ 0.05, Wald-type test) are indicated by different lowercase letters. For all box plots, the central line indicates the median, the bottom and top edges of the box the interquartile range, and the box plot whiskers are plotted down to the minimum and up to the maximum value.

The poor gravitropic response and auxin distribution defects in CA-CPK30 roots prompted us to analyze the expression and localization of the auxin transporters PIN1 and PIN2 *via* whole mount immunolocalization after 2 days induction with 2.5 μM β-estradiol. In CA-CPK30#21, both the anti-PIN1 signal and anti-PIN2 signals were significantly reduced compared to WT (Figures 3A–D). Interestingly, a prominent lateral PIN1 signal, that was not observed in WT, became apparent in the CA-CPK30#21 line (Figures 3A,E), suggesting a defect in PIN1 polarity. We expanded these analysis with other CA-CPK lines to provide a qualitative assessment of effects on PIN levels and polarization in them (Supplementary Figures 3A,B). Additionally, we generated maximal projections of 70 μm z-stacks across the root (Baster et al., 2013), to visualize the overall PIN protein levels in the roots (Supplementary Figure 3C). *Via* these analyses, we found that the reductions in PIN1 and PIN2 protein levels and PIN1 polarization seen in CA-CPK30#21, could also be detected in CA-CPK30#9, CA-CPK13#8 and CA-CPK13#12, with the effects on PIN1 polarization being less prominent in CA-CPK13#8 (Supplementary Figures 3A–C). In addition to the CA-CPK13 and CA-CPK30, both lines for CA-CPK2, CA-CPK4, CA-CPK11, CA-CPK12, and CA-CPK29 displayed clear reductions in PIN signals in their root meristems (Supplementary Figure 3C). The lines with reduced PIN levels largely corresponded with the CA-CPK lines in which root length was reduced (Figure 1N). Additionally, we observed a tendency toward more lateral PIN1 signals in CA-CPK22 (#20 and #15) and in CA-CPK29#10 (but not in #23) (Supplementary Figure 3A), suggesting that these CPKs are also potential regulators of PIN polarity. The reduced PIN1 polarity is consistent with a recent report demonstrating that CPK29 can interact with and phosphorylate PIN proteins (Lee et al., 2021).

Collectively, these data demonstrate that CA-CPK30 impairs not only PIN1 and PIN2 protein levels but also PIN1 polar distribution.

### Constitutive Active CPK30 Impairs Brefeldin A-Sensitive Trafficking

CPK29 was recently shown not only to affect PIN polarity, but also to suppress its internalization rates (Lee et al., 2021). Therefore, we asked if internalization would be affected by CA-CPK30. We estimated PIN internalization rates using a short-term treatment with the fungal toxin Brefeldin A (BFA) that blocks recycling and causes aggregation of the endosomes and their cargoes in so-called BFA bodies (Geldner et al., 2001). Therefore, we simultaneously monitored the localization of PIN1 and the ARF1 GTPase, which labels the Golgi and TGN/EEs (Xu and Scheres, 2005). A 1 h treatment with 25μM BFA induced the formation of BFA bodies in WT roots that were positive for both PIN1 and ARF1 (Figures 3F,G). In contrast, nearly no PIN1 signal was observed in internal structures, but was retained at the plasma membrane, nor were the ARF1-positive endosomes aggregated upon BFA treatment in CA-CPK30#20 (Figures 3F,G). To exclude that this was an artifact of CA-CPK30#21 on ARF1 localization to endosomes, we repeated this dual labeling experiment using anti-CLATHRIN HEAVY CHAIN (CHC) antibodies as a marker of the TGN/EEs (Shimizu et al., 2021). Similarly, CA-CPK30 interfered with the induction of PIN1- and CHC-labeled BFA bodies (Supplementary Figures 4A,B). This suggests that overexpression of CA-CPK30 impairs PIN endomembrane trafficking, and BFA-sensitive aggregation of endosomes.

To better understand this, we screened all our CA-CPK lines for BFA body formation (Supplementary Figures 5A,B). All of the CA-CPK13 and CA-CPK30 lines were almost devoid of PIN1 or PIN2 positive BFA bodies. Also, no BFA bodies could be discerned in CA-CPK4 and CA-CPK11 lines, while all other lines showed some signs of PINs accumulating in BFA bodies, even though their PIN protein levels were also reduced (Supplementary Figure 3C). This indicates that the inhibition of PIN1 accumulation in BFA bodies is not a universal feature of CA-CPKs that cause reduction of PIN protein levels.

### Constitutive Active CPK30 Impacts on Endocytosis and Brefeldin A-Sensitive Trafficking

**FIGURE 4:**
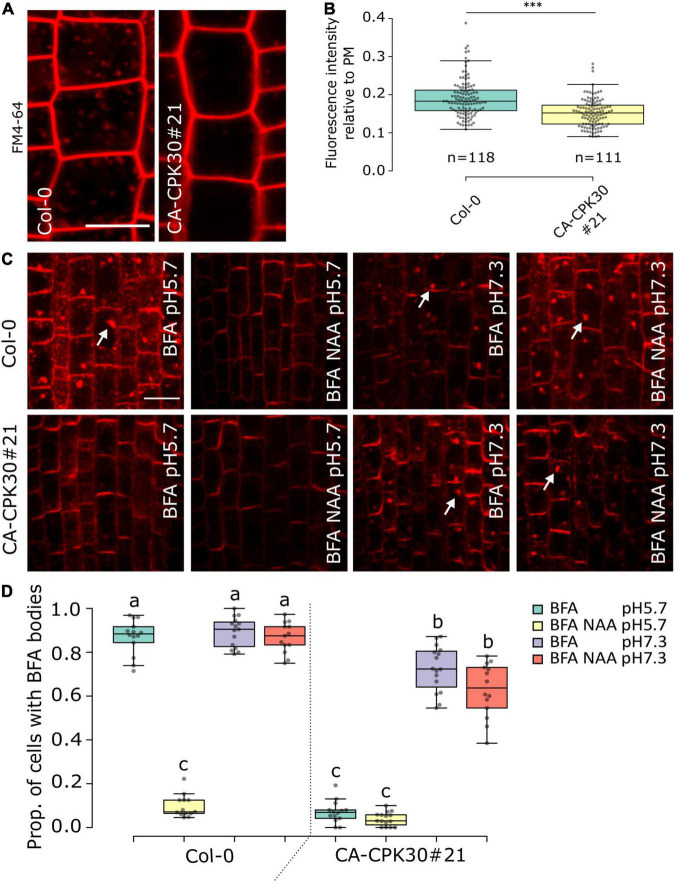
BFA insensitivity in CA-CPK30 depends on the extracellular pH. **(A)** Representative confocal images of FM4-64 uptake in β-estradiol-treated (1 day, 2.5 μM) Col-0 and CA-CPK30#21 seedlings in root epidermal cells. The 5-days-old seedlings were pretreated with liquid medium for 20 min, followed by 10 min incubation in liquid medium containing 5 μM FM4-64. The contrast was equally enhanced in both pictures for illustrational purposes. Measurements were done on the original images without saturation of pixels. **(B)** Quantification of FM4-64 fluorescence intensity in the cytoplasm relative to the plasma membrane of Col-0 and CA-CPK30#21. Numbers (n) represent the cells analyzed from 11 individual seedlings for each line, over three replicates. Unpaired Student’s *t*-test; ^***^P < 0.001. Scale bar = 20 μm. **(C)** Whole-mount immunolocalization using anti-PIN1 antibodies in 5 day-old seedling root meristems of β-estradiol treated wild-type (Col-0) and CA-CPK30#21 seedlings that were treated with BFA or BFA/NAA at pH5.7 and at pH 7.3. Seedlings were transferred for 1 day to 2.5 μM β-estradiol prior to different treatments. Seedlings were incubated in liquid β-estradiol-containing medium with pH 5.7 or pH 7.3 for 30 min then incubated with 25 μM BFA or combined with 10 μM NAA in pH 5.7 or pH 7.3 for 1 h. White arrows indicate PIN1 in BFA bodies. Scale bar = 20 μm. **(D)** Quantification of the proportion of cells with BFA bodies for treatments described in **(C)** (for Col-0: *n* = 14 in BFA, pH5.7; *n* = 13 in BFA/NAA, pH5.7; *n* = 15 in BFA, pH7.3; *n* = 13 in BFA/NAA, pH7.3) and (for CA-CPK30#21: *n* = 14 in BFA, pH5.7; *n* = 15 in BFA/NAA, pH5.7; *n* = 16 in BFA, pH7.3; *n* = 14 in BFA/NAA, pH7.3), three replicates. For the box plots, significant differences (*P* ≤ 0.05, Wald-type test) are indicated by different lowercase letters. The central line indicates the median, the bottom and top edges of the box the interquartile range, and the box plot whiskers are plotted down to the minimum and up to the maximum value.

The agravitropism (Figures 2A,B), ectopic DR5rev::GFP in the LRC (Figures 2C,D), the reduced PIN1 polarization (Figures 3A,E) and the defect in PIN BFA body formation in CA-CPK30 (Figures 3F,G) are reminiscent of phenotypes induced by overexpression of a dominant negative fragment of CHC, which causes defects in clathrin-mediated endocytosis (Kitakura et al., 2011). Therefore, we monitored CA-CPK30’s ability for uptake of the endocytic tracer dye FM4-64 (Figures 4A,B). After 10 min, the FM4-64 stained the plasma membrane and numerous endosomes in WT roots. In contrast, in CA-CPK30#21 nearly no FM4-64 signal could be detected in endosomes, suggesting CA-CPK30 interferes with endocytosis. Importantly, however, our BFA experiments also revealed that CA-CPK30#21 was defective in BFA-induced endosomal aggregation, suggesting that endosomal motility is affected, thereby possibly precluding the efficient movement of FM4-64 positive endosomes into the cell’s interior.

A similar effect on endosomal motility and FM4-64 uptake have previously also been reported for treatments with the protonophore Endosidin9 (Dejonghe et al., 2016) and the synthetic auxin, 1-NAA (Narasimhan et al., 2021). The effects of these molecules on endosomal dynamics could be, at least in part, reverted by increasing the apoplastic pH to reduce their effect on cytoplasmic acidification (Dejonghe et al., 2016; Narasimhan et al., 2021). Therefore, we asked if these effects of CA-CPK30 on BFA body formation could also be reverted at higher pH. We compared BFA body formation at the standard acidic pH 5.7, and the neutral pH7.3 (Figures 4C,D). As a positive control for the assay we used the pH-dependence of 1-NAA on BFA body formation (Narasimhan et al., 2021). The BFA treatment caused accumulation of PIN1 in BFA bodies and could be inhibited by 1-NAA co-treatment at pH 5.7 (Figures 4C,D). In contrast, 1-NAA lost its ability to inhibit PIN1 BFA body formation at pH 7.3 in WT (Col-0) (Figures 4C,D). Similarly, while CA-CPK30 strongly interfered with PIN1 BFA body formation at pH 5.7, it could no longer prevent BFA bodies at pH 7.3 (Figures 4C,D). Even a 1-NAA treatment of CA-CPK30 seedlings could not prevent BFA body formation at pH7.3 (Figures 4C,D). These data indicate that the CA-CPK30 effects on endosomal trafficking are pH-dependent, and thus that the effects on PIN endocytosis and polarity are possibly indirect.

## Discussion

The endomembrane system is undisputedly connected to Ca^2+^. Its membranes separate the cytoplasm from Ca^2+^ that accumulates in lumen of organelles and the apoplast. Every fusion or fission event involves breaching the membrane integrity, during which Ca^2+^ can enter the cytoplasm. It is therefore not surprising that Ca^2+^ was recruited in the regulation of endomembrane trafficking. This is very well established during rapid tip-growth of pollen tubes and root hairs (Himschoot et al., 2017), and this likely also holds true in all cell types. However, very few Ca^2+^ sensors have been characterized as regulators of endomembrane trafficking. One recent example of this is that Ca^2+^ is required for the function of the endocytic TPLATE COMPLEX (Van Damme et al., 2011; Yperman et al., 2021). Here, we found that the Ca^2+^ binding kinase CPK30 can interfere with PIN trafficking, FM4-64 uptake and BFA-sensitive endosomal aggregation, providing a first link between a Ca^2+^ binding kinase and endomembrane trafficking. Notably, biochemical assays have suggested that CPK30 is activated by Ca^2+^ at concentrations that are lower than the cytoplasmic resting Ca^2+^ levels (Boudsocq et al., 2012; Liese and Romeis, 2013), implying its activity would be insensitive to cytoplasmic Ca^2+^ increases. However, CPK30 activity was found to be regulated by Ca^2+^ signals *in planta*, suggesting that CPK30 is kept in a low Ca^2+^ environment at the molecular level to allow activation by Ca^2+^ signals (Liu et al., 2017).

Recently, it was shown that nitrate coordinately stimulates cell elongation and cell proliferation and lateral root development *via* effects on PIN2-mediated auxin transport (Vega et al., 2021; Ötvös et al., 2021). This could be linked to nitrate regulated PIN2 phosphorylation changes that stimulated PIN2 secretion and recycling (Ötvös et al., 2021). We found that CPK30 impacts on root growth responses, and PIN2-mediated auxin transport at least *via* an effect on PIN2 abundance. Therefore, it would be of interest to evaluate if CPK30-like CPKs could directly phosphorylate PINs as part of the root’s nitrate responses.

We noted that induction of CA-CPK30 interfered not only with PIN accumulation in BFA bodies, but rather with BFA-induced endosomal aggregation. A similar phenotype was previously also reported for treatments with the protonophore Endosidin9 (Dejonghe et al., 2016) and the synthetic auxin 1-NAA (Narasimhan et al., 2021). The effects of both treatments could be reverted by increasing the extracellular pH, and are thus linked to induction of cytoplasmic acidification. Such acidification possibly interferes with the protein recruitment to endomembranes *via* negative charges in acidic phospholipids (Himschoot et al., 2017), thereby explaining its disruptive effect on endomembrane processes. We found that the effect of induced CA-CPK30 activity on BFA body formation could also be reverted by increasing the extracellular pH. This suggests that CA-CPK30 inhibits BFA body formation by inducing cytoplasmic acidification, rather than by direct effects on endosomal machinery. Despite the pH-dependence of the BFA-resistance in CA-CPK30, it does not preclude direct effects on other endosomal components, as direct phosphorylation of the endocytic regulator DRP2B was reported for CPK5 (Yip Delormel et al., 2022).

Cytoplasmic acidification implies interference with proton efflux, activation of proton influx, or by a combination of both. Plasma membrane (PM) H^+^ ATPases are prominent phosphorylation-regulated targets for many signaling pathways (Haruta et al., 2015), and could be even inhibited through phosphorylation by the Ca^2+^-regulated PSK5/CIPK11 kinase (Fuglsang et al., 2007). Thus far, CPK30-like CPKs have been shown to inhibit K^+^ influx by direct phosphorylation of the inward rectifying K^+^ channel KAT2 and KAT1 in guard cells (Ronzier et al., 2014), but not in phosphorylation of PM H^+^ ATPases. Yet, a grape berry group I CPK was found to phosphorylate and stimulate H^+^ ATPases (Yu et al., 2006). Therefore, it will be of interest to determine how these group III CPKs could cause cytoplasmic acidification, and whether or not this involves a direct or indirect effect on PM H^+^ ATPase activity.

Our analysis also provide a an entry point into exploring the role and impact of CPK-mediated signaling in the root. While we have focused here on CPK30, it is clear from our phenotypic screen that multiple CPKs control processes that are can modify root growth. While we did not analyze all features of these lines in detail *via* quantifications, we believe that the images can provide clues about other CPKs having a potential role in controlling PIN protein levels, trafficking and polarity. In example, our data indicate that CA-CPK29 has PIN polarity defects, which is consistent with a recent report about CPK29 controlling PIN polarity *via* direct interaction and phosphorylation (Lee et al., 2021). It will therefore be of interest to explore how these different CA-CPK lines lead to defects in PIN protein levels, polarity and/or BFA-sensitive trafficking. This will provide the onset of research into how CPK-mediated signals converge on auxin-regulated root growth.

## Data Availability Statement

The original contributions presented in the study are included in the article and Supplementary Material, further inquiries can be directed to the corresponding author.

## Author Contributions

RW, EH, and JC performed the experiments. RW and SV analyzed the data and drew the figures. MB, DG, JF, TB, and SV planned and designed the study. All authors contributed to the article and approved the submitted version.

## Conflict of Interest

The authors declare that the research was conducted in the absence of any commercial or financial relationships that could be construed as a potential conflict of interest.

## Publisher’s Note

All claims expressed in this article are solely those of the authors and do not necessarily represent those of their affiliated organizations, or those of the publisher, the editors and the reviewers. Any product that may be evaluated in this article, or claim that may be made by its manufacturer, is not guaranteed or endorsed by the publisher.

## Footnotes

1
https://imagej.net/Fiji

2
www.sas.com
