# Corrigendum: Constitutive active CPK30 interferes with root growth and endomembrane trafficking in *Arabidopsis thaliana*

**DOI:** 10.3389/fpls.2022.1100792

**Published:** 2022-12-01

**Authors:** Ren Wang, Ellie Himschoot, Jian Chen, Marie Boudsocq, Danny Geelen, Jiří Friml, Tom Beeckman, Steffen Vanneste

**Affiliations:** ^1^ Department of Plant Biotechnology and Bioinformatics, Ghent University, Ghent, Belgium; ^2^ VIB Center for Plant Systems Biology, Ghent, Belgium; ^3^ Université Paris-Saclay, CNRS, INRAE, Univ. Evry, Institute of Plant Sciences Paris-Saclay (IPS2), Orsay, France; ^4^ Université de Paris, Institute of Plant Sciences Paris-Saclay (IPS2), Orsay, France; ^5^ Department of Plants and Crops, Ghent University, Ghent, Belgium; ^6^ Institute of Science and Technology Austria, Klosterneuburg, Austria; ^7^ Lab of Plant Growth Analysis, Ghent University Global Campus, Incheon, South Korea

**Keywords:** calcium-dependent kinase, CPK30, endosome, Brefeldin A, PIN, root, gravitropism, polarity

In the published article, there was an error in **Figure 1N**. The labels III and IV under CPK28 and CPK8, 13 and 30, respectively, were swapped, thereby not adhering to the commonly used grouping (as depicted in **Figure 1A**) of the CPK family. The corrected **Figure 1** and its caption appear below.

**Figure 1:**
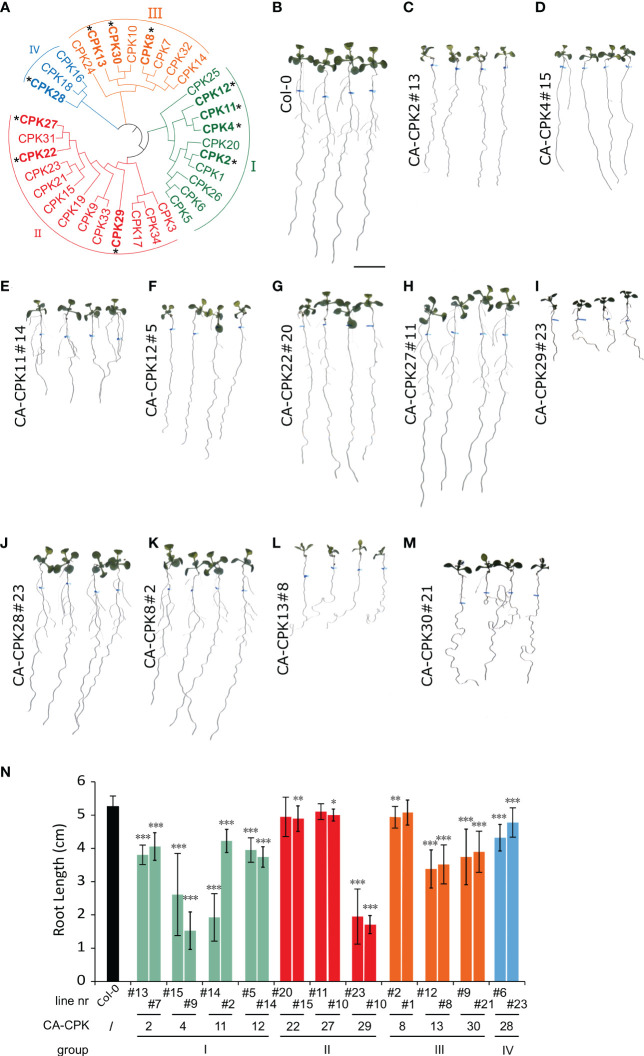
Phenotypic screen of constitutive active CPKs. **(A)** Phylogenetic tree of the Arabidopsis CPK family, with indication of the four main subgroups (I-IV).CPKs indicated with an asterisk were analyzed *via* stable overexpression lines. **(B–M)** Overview of macroscopic phenotypes of CA-CPK lines relative to WT (Col-0).Seeds were grown for 5 days on 1/2 × MS medium then transferred to medium supplemented with 2.5 μM β-estradiol for another 7 days. Scale bar = 1 cm. **(N)** Quantification of root length for indicated CA-CPK lines. Data are represented as the mean ± SD of at two independent replications. *n* ≥ 12 for all lines, except for CPK12#14 (n = 6) and CA-CPK22#20 (*n* = 7). Asterisk indicates significant difference (Unpaired Student’s *t*-test; *P ≤ 0.05, **P ≤ 0.01, ***P ≤ 0.001) between transgenic lines and WT (Col-0) plants.

The authors apologize for this error and state that this does not change the scientific conclusions of the article in any way. The original article has been updated.

## Publisher’s note

All claims expressed in this article are solely those of the authors and do not necessarily represent those of their affiliated organizations, or those of the publisher, the editors and the reviewers. Any product that may be evaluated in this article, or claim that may be made by its manufacturer, is not guaranteed or endorsed by the publisher.

