# Supplementary material for: Constitutive Active CPK30 Interferes With Root Growth and Endomembrane Trafficking in Arabidopsis thaliana
Source: Front Plant Sci. 2022 Jun 16;13:862398. doi: 10.3389/fpls.2022.862398 (PMC9245594; doi:10.3389/fpls.2022.862398)
Supplement: Supplementary file 1 [file Data_Sheet_1.docx]

Supplementary Material

**Supplementary Figure 1| Overview of CA-CPK constructs and root phenotypes of independent CA-CPKs lines.**

**(A)** Scheme of functional domain in CPKs, containing from N- to C-terminus, a variable domain, a kinase domain, an autoinhibitory domain and a calmodulin (CaM)-like domain. Via truncation of the C-terminal domain, constitutive active (CA) CPKs were created.

**(B)** Phenotypic assessment of a second (independent) line, for the CA-CPKs quantified in Figure 1N. Seeds were grown for 5 days on 1/2MS medium then transferred to medium supplemented with 2.5 μM β-estradiol for another 7 days. Scale bar = 1cm.

**Supplementary Figure 2| Analysis of root gravitropic response in DR5rev::GFP background**

1. Gravitropic response of DR5rev::GFP and CA-CPK30#21 x DR5rev::GFP in 5 day old seedlings that were transferred to 2.5 μM β-estradiol medium and immediately gravistimulated for 24h. White arrowheads mark the position of the root tip at the moment of transfer. Scale bar=0.2cm.
2. Quantification of the root angle distribution of (**A**). n is the number of roots analyzed across 2 replicates.

**Supplementary Figure 3| Analysis of PIN1 and PIN2 subcellular localization and levels in different CA-CPK lines.**

**(A-B)** Immunolocalization of PIN1**(A)** and PIN2 **(B)** in roots of Col-0 and two independent CA-lines lines for representatives of the different CPK groups. Group I: CPK2, 4, 11, 12; Group II: CPK22, 27 and 29; Group III: CPK8, 13, 30; Group Ⅳ: CPK28. 5-day-old seedling were transferred to a 2.5µM β-estradiol plate for 1 day. **(C)** Immunolocalization pictures represent maximum intensity projection of 20 3.5 μm Z-sections through the whole root of the immunolocalization experiment described above. Both anti-PIN1 and anti-PIN2 were combined in the immunolocalization protocol, as both proteins are expressed in largely non-overlapping domains. Scalebars in (**A)** and (**B)** = 10 µm and in (**C**) = 20µm.


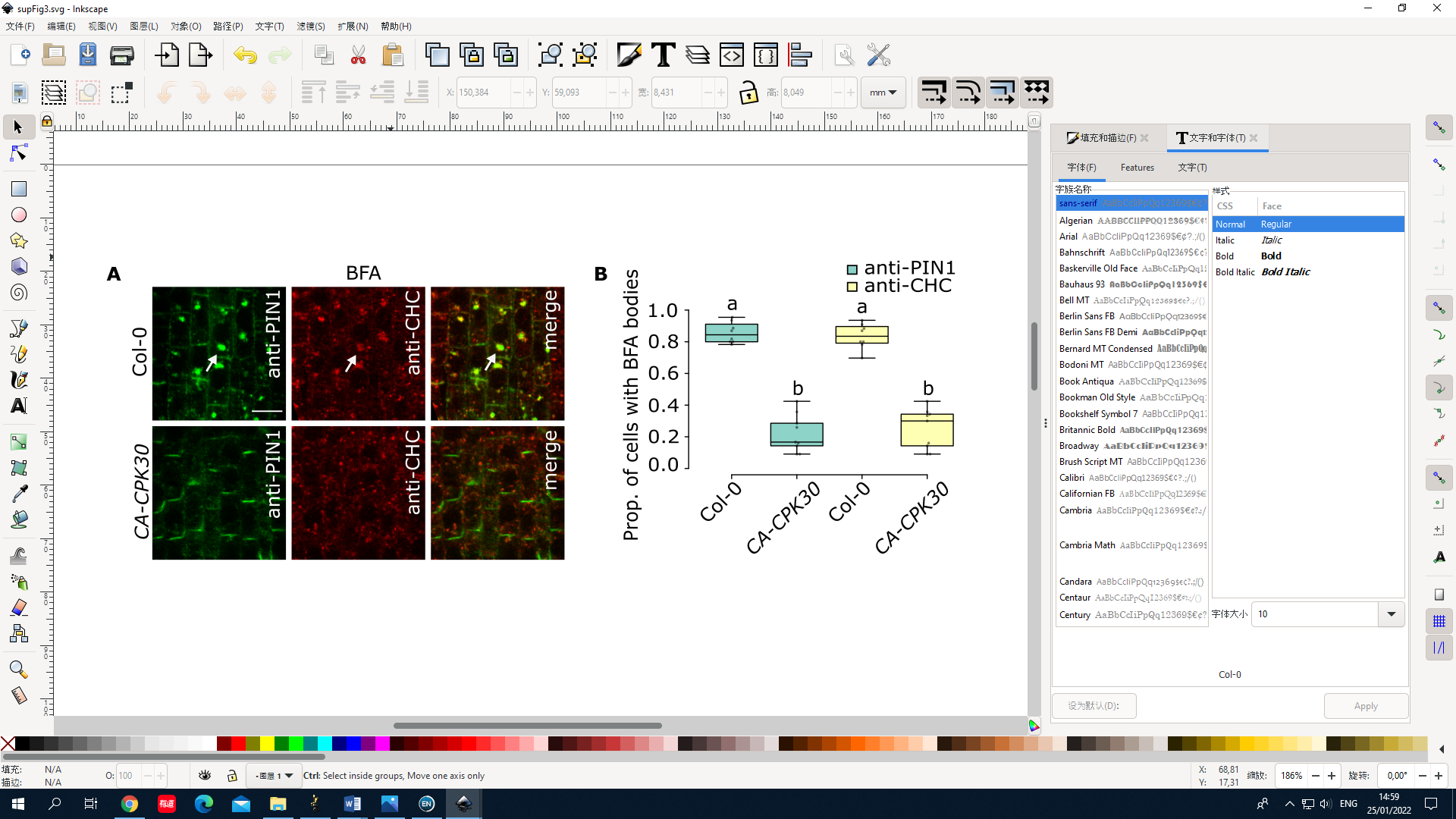


**Supplementary Figure 4| PIN1 and CHC immunolocalization upon induction of Col-0 and CA-CPK30.**

**(A)** Whole-mount immunolocalization using anti-PIN1 and anti-CHC antibodies in 5 day-old seedling root meristems of β-estradiol-treated wild type (Col-0) and CA-CPK30#21 seedlings. Seedlings were transferred for 1 days to 2.5µM β-estradiol prior to BFA treatment. White arrows indicate PIN1, CHC and merged accumulated BFA bodies. Scale bar=10 µm.

**(B)** Boxplot representation of the proportion of cells with BFA bodies for treatments to Col-0 (n=8 in total) and CA-CPK30#21 (n=9 in total) from two replicates.

For the box plots, significant differences (P ≤ 0.05, Wald-type test) are indicated by different lowercase letters. For all box plots, the central line indicates the median, the bottom and top edges of the box the interquartile range, and the box plot whiskers are plotted down to the minimum and up to the maximum value.

.

**Supplementary Figure 5| BFA body formation in Col-0 and CA-CPK lines.**

(A-B) Immunolocalization of PIN1(A) and PIN2 (B) in roots of Col-0 and indicated CA-CPK lines. 5-day-old seedlings were transferred to β-estradiol plates (2.5 µM) for at least 24 h, prior to BFA treatment (1h 25 µM BFA in liquid medium). Scale bar = 10 µm. Arrowheads indicate the presence of BFA bodies. Group I: CPK2, 4, 11, 12; Group II: CPK22, 27 and 29; Group III: CPK8, 13, 30; Group Ⅳ: CPK28.
